# Effect of Post-Weaning Concentrate Feeding Prior to Forage Finishing on Intramuscular Fat Deposition

**DOI:** 10.3390/ani14030496

**Published:** 2024-02-02

**Authors:** Susan K. Duckett, Enrique Pavan

**Affiliations:** 1 Department of Animal and Veterinary Sciences, Clemson University, Clemson, SC 29634, USA; 2 Instituto Nacional de Technolgia Agropecuaria—INTA, Balcarce 7620, Argentina; pavan.enrique@inta.gob.ar; 3 Facultad de Ciencias Agrarias, Universidad Nacional de Mar del Plata, Balcarce 7620, Argentina

**Keywords:** beef, intramuscular fat, carcass quality, forage, concentrates

## Abstract

**Simple Summary:**

Marbling or intramuscular fat deposition in beef is a major determinant of carcass quality and value. Providing high-grain diets during the finishing phase stimulates intramuscular fat deposition, and the carcass quality grade is higher when steers are corn-fed versus grass-fed. This study examined the efficacy of feeding high-concentrate diets for differing times during the post-weaning phase to enhance intramuscular fat deposition and improve carcass quality before forage finishing. Feeding concentrates for 120 day post-weaning enhanced intramuscular fat deposition by altering lipogenic/lipolytic gene expression during the post-weaning phase that was maintained during forage finishing. At slaughter, steers that were fed concentrates for 120 day had higher-quality grades at similar body weights and yield grades than the steers that were fed shorter time periods during the post-weaning phase. The polyunsaturated fatty acid content of the longissimus muscle at slaughter was altered with concentrate feeding post-weaning, but the overall ratio of n-6 to n-3 fatty acids was below the threshold regarded as beneficial for human health.

**Abstract:**

The objectives of this study were to examine the effects of feeding high-concentrate diets post-weaning (PW) prior to forage finishing on (1) changes in ultrasound intramuscular fat deposition and lipogenic/lipolytic gene expression during the post-weaning phase and (2) carcass characteristics and fatty acid composition after forage finishing to 487 kg. Steers were randomly assigned to one of four treatments (PW0, PW40, PW80, and PW120) at weaning to examine the time of high-concentrate feeding prior to forage finishing. The ultrasound intramuscular fat content was greater (*p* < 0.05) for PW120 compared to those for PW0, PW40, or PW80 at the end of the post-weaning phase. Feeding high concentrates (PW120) up-regulated (*p* < 0.01) the mRNA expression of fatty acid transporters and lipogenic genes and down-regulated lipolytic genes in the LM compared to PW0. Carcasses from PW120 were graded 83% Choice (*p* = 0.025), whereas carcasses from other post-weaning treatments (PW0, 40, or 80) were graded 25, 36, and 54% Choice, respectively, at the final harvest. The total fatty acid content of the muscle at slaughter was greater (*p* = 0.0004) for PW120 than PW0, PW40, and PW80. Feeding high-concentrate diets to steers post-weaning for 120 day enhanced early intramuscular fat deposition without causing major changes to the fatty acid composition of the longissimus muscle after forage finishing.

## 1. Introduction

When finished to a similar age endpoint, steers that are forage-finished have lower-quality grades and intramuscular fat content (IMF) compared to their contemporaries that are finished on high-concentrate diets [1,2]. In a review of 15 studies comparing the meat quality of grass-fed and grain-fed beef, grain feeding was related to increased marbling scores in about half of the studies but was variable depending on the finishing endpoint [3]. Research shows that beef flavor is a major factor in the demand for beef [4]. Of the pre-harvest factors that influence beef flavor, the marbling level is the most important factor for beef flavor development in relation to the change in the fatty acid composition [4]. Research indicates that IMF deposition proceeds more rapidly when the carcass weight is around 200 to 400 kg and that the final IMF amount depends on levels attained at the end of the growing period [5,6]. Therefore, more research is needed to explore the early enhancement of IMF deposition and key genes that may be involved in this process.

Previous research has shown that feeding high concentrates post-weaning for 120 d increases marbling scores in steers harvested at that age [7] and after finishing on forages [8]. Early weaning and exposure to a high-concentrate diet has been reported to increase marbling deposition in finished cattle [9], whereas carcass weights are often decreased in turn. Feeding high-starch diets to young calves stimulates intramuscular adipocyte proliferation and alters lipogenic gene expression [10]. The supplementation of high grain during the late gestation and early weaning phases activated lipogenic transcriptome to enhance intramuscular fat deposition [11]. The objectives of this study were to examine the effects of feeding high-concentrate diets post-weaning (PW) prior to forage finishing on (1) the changes in the ultrasound intramuscular fat deposition and lipogenic/lipolytic gene expression during the post-weaning phase and (2) the carcass quality and fatty acid composition after forage finishing.

## 2. Materials and Methods

### 2.1. Animals

Experimental procedures were reviewed and approved by the Clemson University Animal Care and Use Committee, AUP2015-069. Angus cross steers (n = 48) were selected from Clemson University Simpson Research and Education Center after weaning. Steers were blocked by weight and randomly assigned within block to post-weaning (PW) treatments for 0 d (PW0), 40 d (PW40), 80 d (PW80), or 120 d (PW120). Steers were individually fed concentrates using Calan gates and adjusted to the high concentrate ration using a two-step program during the first 21 day of the study (Table 1). Training to Calan gates and adjustment to high-concentrate diets occurred during the treatment phase to avoid any previous supplementation of the steers before the start of this study. No creep feeding was conducted on the calves during the suckling phase.

Then, steers (n = 12/treatment) were fed high concentrates for their assigned treatment periods (PW0, PW40, PW80, or PW120) during post-weaning phase. Steers were weighed and ultrasounded at 40 d intervals during the post-weaning phase and then at 200 d. After steers completed their assigned treatments, they were moved to pasture and grazed high-quality forages (non-toxic tall fescue, rye/ryegrass, oats, and alfalfa) to final live weight of 487 kg. Steers were rotated through pastures at a rate that permitted ample forage to achieve an ADG of 0.62 kg/d or greater throughout the duration of the study. High-concentrate diets had a fatty acid composition of 57.7% linoleic (C18:2 cis-9,12) acid, 26.1% oleic (C18:1 cis-9) acid, and 12.8% palmitic (C16:0) acid, whereas the forage diets contained 57.7% linolenic (C18:3 cis-9,12,15) acid, 18.1% linoleic acid, and 16.3% palmitic acid. When groups reached average target slaughter weight, steers were transported (145 km) to a commercial packing plant for slaughter.

### 2.2. Real-Time Ultrasound

Real-time ultrasound measures of fat thickness, ribeye area, and intramuscular fat percentage were collected at 40–60 d intervals between the 12th and 13th ribs using an Aloka 500-V ultrasound (Corometrics Medical Systems, Wallingford, CT, USA) equipped with a 17 cm, 3.5 MHz linear probe. The images were interpreted using Biosoft Toolbox (Biotronics, Inc., Ames, IA, USA).

### 2.3. Muscle Biopsy

Longissimus muscle needle biopsies were obtained at 120 d from a subset of steers (n = 8 per treatment) from the PW0 and PW120 treatments. Ultrasound IMF values showed separation between PW0 and PW120 starting at 100 d and through 120 d on concentrates, and a subset of steers that represented the treatment mean for each group was biopsied. Steers were shaved, and lidocaine was administered subcutaneously to numb the region to make incision in the hide at the 12th rib. A sterile, disposable biopsy needle (Quick-Core Biopsy Needle, 10G, 10 mm throw length; Cook Medical, Bloomington, IN, USA) was used to obtain longissimus muscle (100 mg) tissue at the 12th rib. Muscle biopsy samples were trimmed of any subcutaneous fat or connective tissue and then immediately frozen in liquid nitrogen. Biopsy samples were transported to Clemson University in liquid N_2_ and stored at −80 °C until extraction.

### 2.4. Relative mRNA Expression

Total RNA was isolated from the biopsy samples using the TriZol procedure (Invitrogen; Thermo-Fisher, Waltham, MA, USA), which included an additional purification step (Pure Link columns, Invitrogen; Thermo-Fisher, Waltham, MA, USA). Total RNA was quantified using a NanoDrop spectrophotometer (Invitrogen) and quality was assessed using Agilent Bioanalyzer (Agilent, Santa Clara, CA, USA). The RNA integrity number (RIN) for all samples was 7 or greater. The RNA (1 μg) was reverse-transcribed in duplicate using qScript (QuantBio, VWR, Atlanta, GA, USA) and used for qPCR. Primer sequences were designed using PrimerQuest (IDT, Coralville, IA, USA), and efficiencies are available in Supplemental Table S1. Several housekeeping genes (glyceraldehyde 3-phosphate dehydrogenase [GAPDH], β-actin [ACTB], and Thy-1 cell surface antigen [Thy1]), ubiquitously expressed prefoldin-like chaperone [UXT], and eukaryotic translation initiation factor 3 subunit K [EIF3K] were evaluated using RefFinder [12]. The most stable housekeeping genes were EIF3K and GAPDH. The geometric mean using EIF3K and GAPDH was calculated and used for qPCR data normalization [13]. Relative gene expression was calculated for each gene compared to PW0 using the 2^−ΔΔCT^ method [14].

### 2.5. Carcass Data

Individual animal identification was maintained throughout the slaughter process. Carcass data were obtained by trained personnel at 24 h postmortem on each individual carcass (steer). At 24 h postmortem, the 10th and 12th ribs from the left side of each carcass were identified, removed, vacuum-packed, and shipped to the Clemson University Meat Laboratory.

### 2.6. Color

Ribs were removed from packaging and allowed to bloom for 15 min. Then, L*, a*, and b* color measurements were taken for the LM and subcutaneous (SQ) fat at the 12th rib. Instrumental color measurements were recorded for L* (lightness), a* (redness), and b* (yellowness) using a Minolta chromameter (CR-310, Minolta Inc., Osaka, Japan) with a 50 mm diameter measurement area using a C illuminant, which was calibrated using the ceramic disk provided by the manufacturer. Values were recorded from three locations of exposed lean and SQ fat at the 12/13th rib interface and averaged.

### 2.7. Adipocyte Histology

During fabrication, a section of subcutaneous tissue (approximately 0.5 cm^2^) was removed external to the 12th rib and embedded in optimum cutting temperature compound in an Intermediate Tissue-Tek cryomold (Sakura Finetek USA, Inc., Torrance, CA, USA). Samples were snap-frozen in liquid N_2_ and stored at −80 °C until analysis. Serial cross-sections (10 μm) were cut through each block at −25 °C using a Microm cryostat (HM 505 E), collected on SuperFrost Plus slides (Fisher Scientific, Fair Lawn, NJ, USA), and fixed in cold acetone for 1 min. Slides (n = 5/carcass) were stained using hematoxylin (Richard-Allan Scientific, San Diego, CA, USA) and eosin-Y (MilliporeSigma, St. Louis, MO, USA) staining procedures. Slides were viewed using an Olympus BX40 (Olympus Corporation, Shinjuku, Tokyo, Japan) with 10× magnification. Pictures were analyzed using Infinity Analyze software version 6.5.0 (Lumenera Corporation, Ottawa, ON, Canada) for volume and perimeter of the subcutaneous adipocytes.

### 2.8. Fatty Acids

The LM was cut into individual 2.54 cm thick steaks for subsequent proximate and fatty acid composition analyses (12th rib), vacuum-packed, and stored frozen at −20 °C until subsequent analyses. Freeze dried samples were transmethylated using sodium methoxide, followed by boron trifluoride [15]. Fatty acid methyl esters were analyzed using an Agilent 6850 gas chromatograph (Agilent, San Fernando, CA, USA) equipped with a flame-ionization detector and Agilent 7673A (Agilent, San Fernando, CA, USA) automatic sampler [16].

### 2.9. Statistics

Data were analyzed in a completely randomized block design using SAS (SAS Inst. Inc., Cary, NC, USA). Steer was the experimental unit. Data were analyzed using the mixed procedure with block and treatment (PW0, PW40, PW80, and PW100) in the model. The percentage data for Choice carcasses in each treatment were analyzed via Chi-squared analyses. For ultrasound measures collected over time, the GLIMMIX procedure was used, and the model included block, treatment, time of measurement, and two-way interaction between time and treatment. Least square means were generated and separated using a protected least significant difference test. Significance was determined at *p* < 0.05.

## 3. Results

During the initial post-weaning treatment period (day 0–40), the PW0 (forage only) steers had a greater (*p* < 0.05) ADG compared to the concentrate-fed steers (PW40, PW80, and PW120) due to an adaptation to the Calan gates and high-concentrate diets for the concentrate-fed steers (Table 2). From day 40 to 80 of treatment, the steers undergoing the PW80 and PW120 treatments had a higher (*p* < 0.05) ADG than those undergoing PW40, which was greater (*p* < 0.05) than PW0. From 80 to 120 day of treatment, the steers undergoing the PW120 treatment had a greater (*p* < 0.05) ADG than those undergoing the PW0 and PW40 treatments, which were greater (*p* < 0.05) than PW80. During the forage-finishing period, the steers’ ADG did not differ between the post-weaning treatments from day 120 to 162 or from 224 to the end of the experiment. From day 162 to 224, the ADG was lower (*p* < 0.05) for PW120 than the other treatments. The overall average daily gain (ADG) was greater (*p* < 0.05) for PW120 than PW80 and PW0, with PW40 being intermediate. The number of days required to reach the target weight endpoint differed (*p* < 0.05), with PW80 requiring a greater number of days compared to the other treatments. The PW40, PW80, and PW120 steers spent 12.5, 22.6, and 36.6%, respectively, after weaning to slaughter on high concentrates.

**Figure 1:**
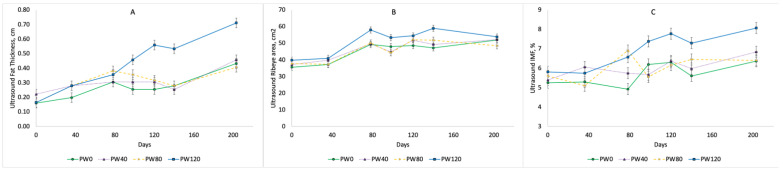
Real-time ultrasound measures of fat thickness (**A**), ribeye area (**B**), and intramuscular fat (IMF; **C**) based on time spent being fed high-concentrate diets during post-weaning phase (PW0, PW40, PW80, PW120) and subsequent forage-finishing phase.

The real-time ultrasound measures for the fat thickness, ribeye area, and intramuscular fat content across time during the post-weaning and forage-finishing phases are shown in Figure 1. The subcutaneous fat thickness was greater (*p* < 0.05) for PW120 compared to PW0, PW40, and PW80 from day 100 to the end of the study. The ultrasound ribeye area was also greater (*p* < 0.05) for PW120 at d 100, 120, and 150 compared to the other treatments; however, the ribeye area estimates were similar at 200 day. The intramuscular fat content, as estimated by ultrasound, was greater (*p* < 0.05) for PW120 at 100, 120, 150, and 200 d compared to those of PW0, PW40, or PW80, which did not differ.

**Figure 2:**
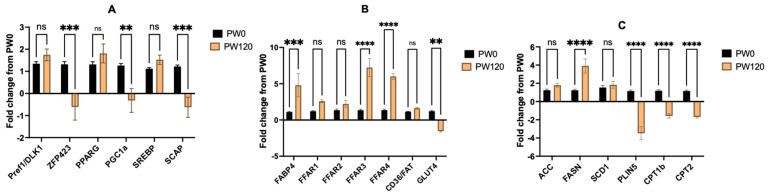
Changes in gene expression of adipogenic genes (**A**), substrate transporters (**B**), and lipogenic/lipolytic genes (**C**) in longissimus muscle biopsies from PW0 and PW120 steers at the end of the 120 d post-weaning phase. **** *p* < 0.0001, *** *p* < 0.001, ** *p*< 0.01, ns = non-significant, *p* > 0.05.

Longissimus muscle biopsies were taken from the steers undergoing the PW0 and PW120 treatments at the end of the 120 d post-weaning phase to examine the changes in gene expression with high concentrate feeding (Figure 2). The expressions of zinc finger protein 423 (ZFP423), peroxisome proliferator-activated receptor gamma (PPARγ), coactivator 1 alpha (PGC1a), and sterol regulatory element-binding protein (SREBP) cleavage activating protein (SCAP) were down-regulated (*p* < 0.01) in PW120 compared to PW0. Th expressions of preadipocyte factor (PREF1/DLK1), PPARγ, and SREBP1c did not differ between PW0 and PW120. Fatty acid binding protein 4 (FABP4) mRNA expression was up-regulated (*p* < 0.0001) in PW120 compared to PW0. Free fatty acid receptors (FFARs) are G protein-coupled receptors (GPR) that facilitate the transport of fatty acids into the cell. Feeding the steers high concentrates during post-weaning (PW120) up-regulated (*p* < 0.01) the mRNA expression of FFAR3 (GPR41) and FFAR4 (GPR120) in the LM compared to PW0. The relative expression of other transporters (FFAR1, FFAR2, and CD36/FAT) were not altered with post-weaning concentrate feeding. Glucose transporter 4 (GLUT4) mRNA expression was down-regulated (*p* < 0.001) at PW120 compared to PW0. Fatty acid synthase (FASN) mRNA expression was up-regulated (*p* < 0.001), whereas the mRNA expressions of lipolytic genes, perilipin 5 (PLIN5), and carnitine palmitoyltransferase (CPT1b and CPT2) were down-regulated (*p* < 0.001) in PW120 compared to PW0. The expressions of other lipogenic genes (SCD1 and ACC) did not differ between PW0 and PW1200.

After forage-finishing to 487 kg BW, the steers were slaughtered and the carcass data were collected (Table 3). The hot carcass weight, fat thickness, marbling score, and quality grades were greater (*p* = 0.036) for PW120 than those for the other post-weaning treatments. Feeding high concentrates to steers for 120 d post-weaning and then finishing them on forages (PW120) produced carcasses that were graded 83% Choice (*p* = 0.025), whereas carcasses from the other treatments were graded 25% (PW0), 36% (PW80), and 54% (PW40) Choice. There were no effects of the post-weaning treatment on the LM or SQ L* or a* or b* measures examined at the end of finishing phase. These results indicate that early post-weaning exposure to concentrates does not influence lean or subcutaneous fat color measurements after finishing on forages.

The histology of the subcutaneous fat samples from the carcasses at slaughter showed greater (*p* < 0.001) areas and perimeters for PW120 than PW80, which were greater (*p* < 0.001) than PW0 and PW40. The adipocyte number did not differ (*p* = 0.106) between the treatments. The total lipid content was greater (*p* < 0.05) and the total moisture content was lower (*p* < 0.05) for PW120 compared to those of the other post-weaning treatments (Table 4). The crude protein content was greater (*p* < 0.05) for PW80 compared to PW0 and PW40, with PW120 being intermediate. The total ash content and individual mineral contents did not differ between the post-weaning treatments.

The fatty acid concentrations of the longissimus muscle (LM) at slaughter are shown in Table 5. The total fatty acid content was greater (*p* = 0.0004) for PW120 than PW0, PW40, and PW80. The myristic (C14:0) acid concentration was greater (*p* < 0.05) for PW120 than PW80 and PW0. The myristoleic (C14:1) acid and trans-10 octadecenoic acid concentrations were greater (*p* < 0.05) for PW120 than all other treatments. The margaric (C17:0) acid concentration was greater for PW120 than PW40 and PW0.

The linoleic (C18:2 cis-9,12) acid and total n-6 polyunsaturated fatty acid (PUFA) concentrations were greater (*p* < 0.001) for PW80 than PW0. In contrast, the linolenic acid (C18:3 cis-9,12,15) concentration was greater (*p* < 0.05) for PW0 than PW40, which was greater (*p* < 0.05) than that of PW120. The eicosapentaenoic (EPA; C20:5) acid concentration was greater (*p* < 0.05) for PW0 than PW40, which was greater (*p* < 0.05) than those of PW80 and PW120. The docosapentaenoic (DPA; C22:5) acid concentration was the highest (*p* < 0.05) for PW0 and the lowest (*p* < 0.05) for PW120. The docosahexaenoic (DHA; C22:6) acid concentration was the highest (*p* < 0.05) for PW0 and the lowest (*p* < 0.05) for PW80. The total polyunsaturated n-3 fatty acid concentration was the highest (*p* < 0.05) for PW0 compared to PW40, which were both greater (*p* < 0.05) than those of PW80 and PW120.

## 4. Discussion

This study was designed to examine the changes in intramuscular fat deposition when feeding steers with high-concentrate diets for differing time periods (PW0, 40, 80, or 120) during the post-weaning phase prior to forage finishing to a 487 kg BW. The overall average daily gain was greater for PW120 than PW80. The PW80 steers had the poorest performance of all treatments and required the greatest number of days to reach the target slaughter weight. These differences appear related to the transition back to forage from concentrates during the day 80 to 120 period, where the gains were about 50% lower than those of PW0 and PW40, and 74% lower than that of PW120 during that time period. Others [7,8,18,19] also reported a higher ADG for steers consuming a high-concentrate-based ration compared to forages. Overall, the steers undergoing the PW120 treatment spent 37% of time during the overall finishing program on concentrates. All steers spent at least 200 day on forage before slaughter.

After 100 d on the concentrate diet, the PW120 steers had greater fat thickness, ribeye area, and intramuscular fat content, as measured by real-time ultrasound, compared to those undergoing the other treatments. These changes in the fat thickness and intramuscular fat deposition continued to the end of the study. The ultrasound ribeye area measures were similar at day 224. These results suggest that at least 100 days of high concentrate feeding after weaning is required to increase the deposition of intramuscular fat in steers prior to forage finishing. These results agree with previous research where steers were fed concentrates for 120 day prior to forage finishing [12,13] and serial slaughter studies that found that 112 day of concentrate feeding was needed to reach low Choice quality grades in yearling steers [20]. All carcasses finished with a yield grade of 1 or 2 in this study, indicating that the early exposure to concentrates followed by forage finishing can produce lean carcasses (yield grade 2 or less). The carcasses from the PW120 treatment were graded over 80% Choice after finishing for 204 on high-quality forages. The treatments with shorter numbers of days on grain (PW0, PW40, and PW80) had lower percentages in the grading Choice (<55%) after finishing on forages. The results of this study demonstrate that steers can be fed high-concentrate diets for 120 d post-weaning to enhance the marbling deposition and produce a high percentage of Choice carcasses after finishing on forages. Pittaluga et al. [21] examined early versus normal weaning on forage- or concentrate-based diets in steers and reported that the marbling score at harvest was not altered by the timing of weaning nor the backgrounding management.

Regarding the expressions of genes involved in adipogenesis, the expressions of ZFP423, PGC1a, and SCAP were down-regulated with the concentrate feeding; however, Pref1/DLK1, PPARg, and SREBP1c expressions were not changed. Pref1 is expressed abundantly in preadipocytes but not in adipocytes and serves as a preadipocyte marker [22]. ZFP423 is expressed in preadipocytes but not in adipocytes and is involved in activating PPARg in undifferentiated cells [23]. A change in ZFP423 but not Pref1 provides mixed results regarding if changes in adipocyte hyperplasia are involved in IMF deposition with concentrate feeding post-weaning. Others [21] have reported that feeding concentrate-based diets during the post-weaning phase altered ZFP423 expression, but these changes were dependent on the time of weaning. Early-weaned steers had a greater expression of ZFP423 during the backgrounding phase (d112), whereas normal-weaned steers had a greater expression of ZFP423 during the finishing phase (d 255). It was also observed that DLK1 expression was greater for steers that were backgrounded on concentrate-based diets than forages early in the backgrounding phase but not in the finishing phase [21]. The expression of transcription factors (PPARg and SREBP1c) was not altered, but their activators (PGC1a and SCAP) were down-regulated with concentrate feeding (PW120) compared to PW0. In the subcutaneous adipose tissue, the number of adipocytes was unchanged with post-weaning concentrate feeding; however, the perimeter and area of the adipocytes increased with concentrate feeding, which demonstrates that the hypertrophy of the adipocyte and filling of lipid was most important in subcutaneous adipose tissue. Robelin [24] examined changes in adipocyte hyperplasia and hypertrophy during growth from 15 to 65% of mature weight. Increases in the subcutaneous fat deposition were accompanied by large changes in hypertrophy (12-fold increase in volume) and smaller changes in hyperplasia (4.6-fold increase in number) throughout growth.

The greatest changes in gene expression were observed for fatty acid transporters. FABP4 expression was up-regulated during adipocyte differentiation [25] and serves as a marker of differentiation in bovine adipocytes [26,27]. FABP4 is a transporter protein that transports intracellular fatty acids to the nucleus, where it alters the transcription of certain genes [27]. Others have reported that FABP4 was associated with subcutaneous fat thickness and marbling in Wagyu x Limousin F_2_ cattle [28]. Guo and co-workers [29] related gene expression to the IMF percentage in cattle and sheep, and they identified FABP4 as having a significant association with IMF in both species. The up-regulation of FABP4 with high-concentrate feeding indicates that exposure may promote the differentiation of preadipocytes and the uptake of long-chain fatty acids into the cell. In addition, FFAR3 (GPR41) and FFAR4 (GRP120) were also up-regulated (*p* < 0.05) for PW120 compared to PW0. In our study, we did not observe any changes in the expression of FFAR1 (GPR40) or FFAR2 (GPR42). Free fatty acid receptor 4 has a high affinity for myristic, palmitic, stearic, and oleic acids and is activated by PUFAs, both n-6 and n-3 [30]. The use of agonists for FFAR4 has shown reductions in non-esterified fatty acids in mice [31], indicating its involvement in the feedback regulation of lipolysis. The activation of FFAR4 has been shown to increase adipogenesis and lipogenesis, improve insulin sensitivity, and reduce inflammation [30,31,32,33,34,35]. In contrast, FFAR3 (GPR41) is a short-chain fatty acid receptor that has a high affinity for propionate, butyrate, and acetate [32]. The up-regulation of free fatty acid receptors (FFAR3 and FFAR4) and FABP4 suggests that the transport of fatty acids to and from the adipocyte may be key to increasing intramuscular lipid deposition in young steers.

The lipogenic gene (FASN) was up-regulated, and lipolytic genes (PLIN5, CPT1b, and CPT2) were down-regulated with post-weaning high-concentrate feeding (PW120) compared to PW0. FASN is a key enzyme for de novo fatty acid synthesis in the adipocyte. Previous research has also shown that increasing the time spent feeding on concentrates up-regulates the lipogenic genes involved in de novo fatty acid synthesis (FASN) and down-regulates the lipogenic genes involved in fatty acid oxidation (PLIN5) [7]. PLIN5 is present in skeletal muscle and localizes to the lipid droplet and mitochondria to shuttle fatty acids for oxidation [36]. The overexpression of PLIN5 alters the expression of genes involved in fatty acid oxidation [36]. In humans, the redistribution of PLIN5 to the lipid droplet increases intramyocellular lipid deposition in skeletal muscle [37]. The down-regulation of PLIN5, CPT1b, and CPT2 with concentrate feeding suggests reduced fatty acid oxidation in the longissimus muscle for PW120 compared to PW0. Other authors have identified PLIN5 in a genome-wide association study in Japanese Black cattle with high intramuscular fat deposition [38]. Alterations in lipolytic gene expression may play a role in enhancing marbling deposition and deserves further investigation.

Feeding concentrates for 120 day post-weaning also increased the total lipid content and total fatty acid content in the longissimus muscle at slaughter by 95% compared to forage-only feeding (PW0). Other authors also reported that cattle consuming a high-concentrate-based diet have greater lipid deposition within the LM in comparison to forage-fed beef cattle [1,2,39]. There were no differences in the ash or mineral content between the treatments, which agrees with previous reports [1,2,39] between forage and concentrate-fed beef steers. The changes in the fatty acid composition with post-weaning concentrate feeding are related to polyunsaturated fatty acid (PUFA) n-6 and n-3 concentrations. The linoleic acid concentration increased, whereas the linolenic and long-chain n-3 fatty acid concentrations all decreased with concentrate feeding during the post-weaning phase. Duckett et al. [20] reported similar reductions in PUFA concentrations with high-concentrate feeding during the finishing phase, indicating that grain feeding during backgrounding or finishing both reduce PUFA concentrations. In comparison between forage- and grain-finishing systems, the major changes in the fatty acid composition are increased oleic acid and total MUFA concentrations and reduced n-3 PUFA [1,19,39,40]. These changes in the individual PUFA content resulted in an increase in the ratio of n-6 to n-3 PUFA with increased post-weaning concentrate feeding; however, all ratios were below 4, which is considered beneficial for human health [41]. Beef flavor is related to the fatty acid composition of the meat, and reductions in n-3 PUFA alter oxidation and the formation of volatile flavor compounds that are attributed to grassy flavor notes [4].

## 5. Conclusions

Feeding high-concentrate diets to steers post-weaning for 120 days prior to forage finishing enhances marbling deposition and the percentage of carcasses being graded Choice without major changes in the fatty acid composition of the muscle. Feeding concentrates during post-weaning up-regulated the expression of fatty acid transporters (FABP4, FFAR3, and FFAR4) that regulate fatty acid movement to and from the adipocyte and down-regulated the lipolytic genes (PLIN5, CPT1b, and CPT2) that regulate fatty acid oxidation in the muscle.

## Figures and Tables

**Table 1:** Dry matter formulation and nutrient composition of high-concentrate-based diets provided during post-weaning phase.

Ingredient/Composition	Starter	Finisher
Chopped hay, %	15.00	5.00
Cracked corn, %	56.50	71.50
Corn gluten feed, %	25.00	20.00
Mineral Premix ^1^, %	3.50	3.50
NEm, Mcal/kg	1.91	2.05
NEg, Mcal/kg	1.28	1.40
Crude Protein, %	13.0	12.5
Crude Fat, %	3.79	3.99
Crude Fiber, %	8.77	5.30

^1^ Mineral Premix: Rumensin^®^ included, 30 g/ton.

**Table 2:** Average daily gain and time required to reach 487 kg live weight in steers that were fed high-concentrate diets for differing periods of time post-weaning and then forage-finished.

	Post-Weaning Concentrate Feeding, Day				
Variable	PW0	PW40	PW80	PW120	SEM
n	12	12	12	12	
Age at start, day	200	200	204	200	4.13
Period weights, kg					
day 0	239	235	237	234	3.52
day 40	278 ^a^	246 ^b^	242 ^b^	244 ^b^	3.56
day 80	308	295	307	302	5.43
day 120	340 ^b^	330 ^b^	323 ^b^	371 ^a^	6.87
day 162	386 ^ab^	379 ^b^	368 ^b^	406 ^a^	7.67
day 224	424 ^a^	413 ^ab^	403 ^b^	428 ^a^	6.80
Final BW, kg	484	484	476	497	7.49
Period ADG, kg/d					
day 0–40	0.92 ^a^	0.32 ^b^	0.20 ^b^	0.28 ^b^	0.092
day 40–80	0.76 ^c^	1.19 ^b^	1.52 ^a^	1.43 ^a^	0.082
day 80–120	0.53 ^b^	0.60 ^b^	0.31 ^c^	1.18 ^a^	0.063
day 120–162	1.13	1.16	1.07	0.84	0.11
day 162–224	0.60 ^a^	0.55 ^a^	0.56 ^a^	0.35 ^b^	0.037
day 224—slaughter	0.66	0.76	0.63	0.70	0.053
Overall ADG, kg/d	0.76 ^ab^	0.78 ^ab^	0.70 ^b^	0.82 ^a^	0.027
Days to target weight	318 ^b^	321 ^b^	344 ^a^	325 ^b^	4.97
Time on forages, d	318 ^a^	281 ^b^	264 ^c^	205 ^d^	5.87
Time on concentrates, d	0 ^d^	40 ^c^	80 ^b^	120 ^a^	2.33

^abcd^ Means with uncommon superscripts in the same row differ (*p* < 0.05).

**Table 3:** Carcass characteristics at slaughter of steers that were fed concentrates post-weaning (PW) for various times and then forage-finished.

	Post-Weaning Concentrate Feeding, Day				
Carcass Measures	PW0	PW40	PW80	PW120	SEM
n	12	12	12	12	
Carcass Traits					
Hot carcass weight, kg	250.0 ^b^	249.3 ^b^	251.5 ^b^	265.6 ^a^	4.40
Dressing percent	53.9	53.7	55.4	55.9	0.64
Fat thickness, cm	0.42 ^b^	0.37 ^b^	0.44 ^b^	0.57 ^a^	0.052
Ribeye area, cm ^1^	68.71	68.55	64.79	74.71	2.42
Kidney, pelvic, and heart fat, %	1.83	1.92	1.97	2.08	0.12
Yield grade	1.9	1.9	2.2	2.0	0.12
Skeletal maturity ^1^	150	155	167	158	4.10
Marbling score ^1^	464 ^b^	461 ^b^	478 ^b^	573 ^a^	18.07
Quality grade ^1^	3.8 ^b^	3.9 ^b^	4.3 ^b^	5.4 ^a^	0.30
Choice, %	25 ^b^	54 ^b^	36 ^b^	83 ^a^	13.76
Carcass Color					
Longissimus L*	44.39	43.84	43.70	42.95	0.62
Longissimus a*	28.78	28.44	29.73	28.86	0.50
Longissimus b*	11.25	10.94	11.32	11.20	0.24
Subcutaneous L*	81.93	83.05	82.29	81.99	0.51
Subcutaneous a*	6.79	6.09	7.10	6.93	0.45
Subcutaneous b*	19.88	18.03	18.30	18.26	0.61
Subcutaneous Adipocyte					
Adipocyte number	282	265	222	217	21.7
Area, μm^2^	5416 ^c^	5333 ^c^	6467 ^b^	6699 ^a^	281.7
Perimeter, um	327 ^c^	321 ^c^	353 ^b^	366 ^a^	9.02

^1^ Code = skeletal maturity: 100 = A, 200 = B; marbling score: 300 = traces, 400 = slight, 500 = small, 600 = modest; quality grade: 3 = Select−, 4 = Select+, 5 = Choice−, 6 = Choice° [17]. ^abc^ Means with uncommon superscripts in the same row differ (*p* < 0.05).

**Table 4:** Proximate analysis of longissimus muscle (LM) at slaughter from steers with increasing time spent being fed concentrates post-weaning and then forage-finished to 487 kg live weight.

	Post-Weaning Concentrate Feeding, d				
	PW0	PW40	PW80	PW120	SEM
Proximate composition, g/100g LM					
Moisture, %	75.38 ^a^	75.04 ^ab^	74.44 ^b^	73.45 ^c^	0.31
Crude protein, %	20.25 ^b^	20.46 ^b^	21.13 ^a^	20.57 ^ab^	0.20
Crude lipid, %	2.07 ^b^	2.45 ^b^	2.49 ^b^	4.27 ^a^	0.29
Ash, %	0.96	0.95	0.94	0.95	0.010
Minerals, mg/100g LM					
Phosphorus	181.1	178.6	177.0	176.2	5.04
Potassium	359.0	355.2	358.5	339.4	9.84
Calcium	11.41	10.52	10.43	9.68	0.70
Magnesium	21.00	20.77	20.63	20.10	0.60
Zinc	2.84	2.70	3.00	2.71	0.11
Iron	1.60	1.57	1.63	1.63	0.072
Sodium	33.51	32.96	32.76	32.74	1.002

^abc^ Means with uncommon superscripts in the same row differ (*p* < 0.05).

**Table 5:** Fatty acid concentration (%) of LM at slaughter of steers that were fed concentrates post-weaning (PW) for differing times and then forage-finished to 487 kg live weight.

	Post-Weaning Concentrate Feeding, Day				
Fatty Acid, %	PW0	PW40	PW80	PW120	SEM
n	12	12	12	12	
C14:0	2.31 ^b^	2.56 ^ab^	2.38 ^b^	2.71 ^a^	0.010
C14:1c9	0.41 ^b^	0.44 ^b^	0.46 ^b^	0.59 ^a^	0.040
C15:0	0.46	0.47	0.48	0.50	0.029
C16:0	26.20	27.22	27.03	27.95	0.44
C16:1c9	3.27	3.14	3.15	3.34	0.13
C17:0	1.08 ^b^	1.15 ^b^	1.21 ^ab^	1.24 ^a^	0.034
C18:0	16.63	16.58	16.43	15.53	0.33
C18:1t9	0.145	0.00	0.095	0.074	0.042
C18:1t10	0.010 ^b^	0.012 ^b^	0.073 ^b^	0.58 ^a^	0.094
C18:1t11	1.88	1.94	2.45	1.96	0.12
C18:1c9	36.61	35.34	35.55	36.29	0.57
C18:1c11	1.30	1.28	1.32	1.38	0.40
C18:2c9,12	2.31 ^b^	2.62 ^b^	3.36 ^a^	3.09 ^ab^	0.20
C18:2c9,t11	0.35	0.39	0.44	0.42	0.030
C18:2t10,c12	0.028	0.036	0.034	0.041	0.0057
C18:3 n-3	0.90 ^a^	0.76 ^b^	0.68 ^bc^	0.57 ^c^	0.048
C20:4 n-6	1.06	1.18	1.27	0.94	0.10
C20:5 n-3	0.46 ^a^	0.35 ^b^	0.22 ^c^	0.14 ^c^	0.029
C22:5 n-3	0.72 ^a^	0.61 ^ab^	0.48 ^b^	0.32 ^c^	0.043
C22:6 n-3	0.10 ^a^	0.090 ^ab^	0.060 ^c^	0.062 ^bc^	0.010
Total Fatty Acids	1.61 ^b^	1.92 ^b^	2.13 ^b^	3.08 ^a^	0.23
Saturated	45.14	46.36	45.84	46.20	0.57
Odd-Chain	1.54	1.62	1.69	1.74	0.054
Monounsaturated	40.28	39.22	39.16	40.23	0.59
Polyunsaturated n-6	3.37 ^b^	3.80 ^ab^	4.63 ^a^	4.02 ^ab^	0.30
Polyunsaturated n-3	2.19 ^a^	1.81 ^b^	1.44 ^c^	1.09 ^c^	0.12
Ratio, n-6:n-3	1.55 ^d^	2.10 ^c^	3.24 ^b^	3.94 ^a^	0.159

^abcd^ Means with uncommon superscripts in the same row differ (*p* < 0.05).

## Data Availability

The data presented in this study are available on request from the corresponding author. The data are not publicly available due to restrictions.

## Supplementary Materials

The following supporting information can be downloaded at https://www.mdpi.com/article/10.3390/ani14030496/s1, Table S1: Primers used for mRNA real-time qPCR.
