# Supplementary material for: Effect of Post-Weaning Concentrate Feeding Prior to Forage Finishing on Intramuscular Fat Deposition
Source: Animals (Basel). 2024 Feb 2;14(3):496. doi: 10.3390/ani14030496 (PMC10854609; doi:10.3390/ani14030496)
Supplement: Supplementary file 1 [file animals-14-00496-s001.zip › animals-2794319-supplementary.pdf]

Supplemental Table S1. Primer sequences (5' to 3') for quantitative real-time PCR of lipogenic genes.

Gene[1]	Forward	Reverse	Efficiency
ACC	AGCTGAATTTTCGCAGCAAT	GGTTTTCTCCCCAGGAAAAG	1.07
ACTB	CTCTTTCAGCCTTCCTTCCT	GGGCAGTGATCTCTTTCTGC	1.01
AMPK	TTGGCACCGATGTAACTTGA	CTGGACAGAAGGAAGCAAGG	1.03
CD36	GCATTCTGAAAGTGCGTTGA	CGGGTCTGATGAAAGTGGTT	1.02
CPT1b	GCACCTCTTCTGCCTTTACG	CGATCTGGCTAGTGGAGAGG	1.00
CPT2	CCTTCCTTCCTGTCTTGGTATG	GGTCTGGGTAAACGAGTTGAA	1.05
EIF3K	CTGACAGACAGCCAGCTAAA	CACGATGTTCTTGGGCTTTATG	1.02
ELOVL5	GTCATCTGGCCGTGTACCTT	GGGAAGAAAAGCTGCTGATG	1.02
ELOVL6	GGAAAGCAACGAAAGCTGAC	TGGGTTGTGTGTTTGCTCAT	1.05
FABP4	CATCTTGCTGAAAGCTGCAC	AGCCACTTTCCTGGTAGCAA	1.00
FASN	GCATCGCTGGCTACTCCTAC	GTGTAGGCCATCACGAAGGT	0.95
FFAR1	TTAGCCTCTCGCTTCTGCTC	GCCTTTAGCTTCCGTCTGTG	1.03
FFAR2	TGGGTCATGTCTTTTGGTCA	GCTCTTGGGTGAAGTTCTCG	1.04
FFAR3	ACTCCTTCTTCCTCGGCAAT	AGATCCGAGAGGGTGAGGTT	1.05
FFAR4	AGGAACGAATGGAGGAGGTT	GTGCTGAGGGTCATGGAAAT	1.04
GAPDH	GGGTCATCATCTCTGCACCT	GGTCATAAGTCCCCTCCACGA	0.97
GLUT4	ACCTTATGGCCACTCCTCCT	CTCAGCCAACACCTCAGACA	0.99
PLIN5	AAGTCAGAGGAGCTGGTGGA	CCCAGAGAGTGCTCATAGGC	1.00
PPARG	AGGATGGGGTCCTCATATCC	GCGTTGAACTTCACAGCAAA	1.00
PPARGC1A	CTTCCTCCTGACACCCATGT	CGCTCCTCAGAAAGAACCAC	0.98
PREF1	TCTGCGCTACAACCACATGT	TGGTGAAGGTGGTVATGTCG	0.98
SCAP	GGCTGATCCATGGTCACTTT	AGTGGGTAGCAGCAGGCTAA	1.05
SCD1	TTATTCCGTTATGCCCTTGG	GGTAGTTGTGGAAGCCCTCA	0.95

SREBP1c	CTGGAGAAGCTGGACTGAGG	GCTTTCCCAAGACTCAGCAC	0.95
Thy1	CACCTCTGCCAATACCACCT	ATACCCCTCCATCCTTCCAC	1.04
UXT	GGTGGATTTGGGCTGTAACT	TGTGGATATGGGCCTTGATATTC	1.00
ZFP423	GAAGACCATCCACGCAGATAAG	GATGCTGCCAAACTGCATTAC	1.00

[1]Acetyl CoA carboxylase (ACC); beta-actin (ACTB); AMP-activated protein kinase (AMPK); cluster differentiation factor 36 or fatty acid translocase (CD36); carnitine palmitoyltransferase 1b (CPT1b); carnitine palmitoyltransferase 2 (CPT2); eukaryotic translation initiation factor 3 (EIF3K); Fatty acid elongase 5 (ELOVL5); fatty acid elongase 6 (ELOVL6); fatty acid binding protein 4 (FABP4); fatty acid synthase (FASN); free fatty acid receptor 1-4 (FFAR1-4); glyceraldehyde 3-phosphate dehydrogenase (GAPDH); glucose transporter 4 (GLUT4); Perilipin 5 (PLIN5); peroxisome proliferator-activated receptor γ (PPARγ); PPARγ coactivator-1 α (PPARGC1A); SREBP cleavage activating protein (SCAP); stearoyl CoA desaturase-1 (SCD); sterol regulatory element-binding protein 1c (SREBP1c); Thy1 cell surface antigen (Thy1); Ubiquitously expressed prefoldin like chaperone (UXT); zinc finger protein 423 (ZFP423).
